# Establishment of a CALU, AURKA, and MCM2 gene panel for discrimination of metastasis from primary colon and lung cancers

**DOI:** 10.1371/journal.pone.0233717

**Published:** 2020-05-29

**Authors:** Parinaz Nasri Nasrabadi, Zahra Nayeri, Ehsan Gharib, Reza Salmanipour, Fatemeh Masoomi, Forouzandeh Mahjoubi, Alireza Zomorodipour

**Affiliations:** 1 Department of Molecular Medicine, Institute of Medical Biotechnology, National Institute of Genetic Engineering and Biotechnology, Tehran, Iran; 2 Basic and Molecular Epidemiology of Gastrointestinal Disorders Research Center, Research Institute for Gastroenterology and Liver Diseases, Shahid Beheshti University of Medical Sciences, Tehran, Iran; 3 Department of Medical Genetics, Institute of Medical Biotechnology, National Institute of Genetic Engineering and Biotechnology, Tehran, Iran; Virginia Commonwealth University, UNITED STATES

## Abstract

Metastasis is known as a key step in cancer recurrence and could be stimulated by multiple factors. Calumenin (CALU) is one of these factors which has a direct impact on cancer metastasis and yet, its underlined mechanisms have not been completely elucidated. The current study was aimed to identify CALU co-expressed genes, their signaling pathways, and expression status within the human cancers. To this point, CALU associated genes were visualized using the Cytoscape plugin BisoGenet and annotated with the Enrichr web-based application. The list of CALU related diseases was retrieved using the DisGenNet, and cancer datasets were downloaded from The Cancer Genome Atlas (TCGA) and analyzed with the Cufflink software. ROC curve analysis was used to estimate the diagnostic accuracy of DEGs in each cancer, and the Kaplan–Meier survival analysis was performed to plot the overall survival of patients. The protein level of the signature biomarkers was measured in 40 biopsy specimens and matched adjacent normal tissues collected from CRC and lung cancer patients. Analysis of CALU co-expressed genes network in TCGA datasets indicated that the network is markedly altered in human colon (COAD) and lung (LUAD) cancers. Diagnostic accuracy estimation of differentially expressed genes showed that a gene panel consisted of CALU, AURKA, and MCM2 was able to successfully distinguish cancer tumors from healthy samples. Cancer cases with abnormal expression of the signature genes had a significantly lower survival rate than other patients. Additionally, comparison of CALU, AURKA, and MCM2 proteins between healthy samples, early and advanced tumors showed that the level of these proteins was increased through normal–carcinoma transition in both types of cancers. These data indicate that the interactions between CALU, AURKA, and MCM2 has a pivotal role in cancer development, and thereby needs to be explored in the future.

## Introduction

Calumenin (CALU) is a known member of the CREC protein family. It is encoded from the 7q32 region of the human genome and translated into a 315 amino acid protein containing a common CREC signal sequence, a putative N-glycosylation region, 6 ~ 7 EF-hand motifs, and a carboxyl-terminal with an inefficient retention HDEF signal sequence [1]. Currently, a total of 15 CALU isoforms have been identified in human [2]. Unlike the other CREC family members, CALU proteins can only be transported via cellular secretory mechanisms, and most of the CALU isoforms (CALU 1–14) are detected in the endoplasmic reticulum (ER), Golgi apparatus and extracellular medium [3–5]. However, CALU-15, which lacks a signal peptide sequence, can only be transported between cellular cytosol and nucleus [2].

CALU isoforms show distinctive roles in cellular functions. As chaperone proteins, CALU isoforms participate in protein correct structure modeling and maturation [3–5]. The implication of CALU function results in the activation of ER-located enzymes such as the γ-carboxylase [6, 7]. Also, CALU can modulate the cellular stress through regulation of GRP78 and phosphorylated PERK as ER-stress factors, C/EBP homologous protein (CHOP), and p-JNK proapoptotic proteins, and antiapoptotic Bcl-2 [3]. Elevation of CALU extracellular isoforms ameliorates SEPT1 expression and increases cell cycle modulation [8].

The upregulation of CALU, however, has been reported to associate with the elevation of cell migration and metastasis in lung and colon cancers. Analysis of human lung cancer patients indicated a higher level of CALU and Oxysterol binding protein-like 5 (OSBPL5) in metastatic cancer cells than non-metastatic samples [9]. Interestingly, cancer cells were shown to increase the CALU secretion from the cancer-associated fibroblasts (CAFs) via a TGF-β induced miR-21 expression axis and promoted tumoral proliferation and metastasis [10]. A high-throughput proteomic study on tumoral biopsies obtained from colon cancer patients detected the co-progressive expression of CALU and Biglycan, as the new biomarkers of colon tumors [11]. This investigation was in line with the previous study of identifying the potential biomarkers of colorectal carcinogenesis, in which reported the CALU as one the metastasis-related proteins upregulated from adenoma to carcinoma [12]. These findings brought us to identify the CALU associated genes network with Bioinformatics tools. Moreover, we developed a signature gene panel consisted of CALU and other differentially expressed genes (DEGs) between healthy and cancer datasets retrieved from the Cancer Genome Atlas (TCGA), and estimated the diagnostic and prognostic performances of this panel in colon and lung cancer patients.

## Materials and methods

### Network visualization and analysis

Network illustration was carried out using the Cytoscape version 3.7.1 [13]. Cytoscape plugin BisoGenet was applied to visualize CALU interactions with neighbored genes [14]. The topology of the network was determined with the Cytoscape plugin CytoHubba version 0.1 [15]. The Gene Ontology (GO) biological process and KEGG pathways annotation were analyzed with the Enrichr web-based application (http://amp.pharm.mssm.edu/Enrichr/). The results were assumed as statistically significant if *P* < 0.05.

### Identification of calumenin related diseases

The list of CALU related diseases was retrieved using the Cytoscape plugin DisGenNet, a bioinformatics platform that integrates the genes data of various human disorders [16]. Following query terms were chosen as data sources: 1- Online Mendelian Inheritance in Man (OMIM) (Mendelian Inheritance in Man and its online version, OMIM), 2- Genetic Association Database (GAD) [17], 3- Mouse Genome Database (MGD) [18], 4- Comparative Toxicogenomics Database (CTD) [19], 5- PubMed, and 6- Uniprot. Ranking of the associations between CALU and diseases was performed according to the number of sources, organism type, and the number of supported publications.

### Sample size estimation

Sample size was determined by using of the chi-square test based on the α error = 0.05 and β error = 0.2 [20]. Analysis of the gene panel expression in the colon cancer training cohort resulted in a proportion of 0.41 in healthy samples, and 0.5 in CRC tumors. The values for the control group and lung tumors in the training set of lung cancer were 0.57 and 0.71, respectively. The estimated ratio between the healthy controls and cancer cases was set at 1:1.2 for colon cancer, and 1:1.25 for lung cancer. On the other hand, due to the existence of one confounder (gene panel), the sample size of the validation set was increased by about 10%, in both types of cancers.

### Data mining and processing

Illumina Hiseq 2000 RNA-seq datasets (level 3 per-gene RNA-seq v2 expression data) were retrieved from TCGA (https://cancergenome.nih.gov). The dbGaP accession number to the specific version of the TCGA datasets is phs000178.v10.p8. The read counts of these datasets were estimated by RSEM package (RNA-seq by expectation maximization). To identify the miRNAs, small reads (< 75 bp) were analyzed with the RNA family algorithms (Rfam, version 13.0, http://rfam.xfam.org), to remove the known small noncoding RNAs including rRNAs, tRNAs, snRNAs, and snoRNAs. The filtered sequences were subsequently analyzed with MIREAP (https://sourceforge.net/projects/MIREAP, 2018), and miRNAs were detected based on their canonical hairpin structure. To identify the protein-coding genes reads > 100 bp were analyzed the Coding‐Noncoding‐Index (CNI, version 2) based on 64 triplets of nucleotides algorithms [21], and additionally examined with the coding potential calculator‐2 (CPC-2) webserver to distinguish noncoding transcripts from mRNAs based on the length and quality rate of the open reading frame of protein-coding transcripts [22]. Next, we analyzed the retrieved protein sequences with the Pfam‐scan (version 1.3) database archive to evaluate their annotation [23], and also used the phylogenetic codon substitution frequency (phyloCSF, release 20121028) to distinguish the coding transcripts from noncoding alignments according to the evolutionary preservation of amino acids with known families of proteins [24]. To identify the human transcription factors and kinases, the coding protein list was uploaded into the Ensembl BioMart web process version 79 (https://www.ensembl.org/Biomart) and analyzed with the human genome assembly GRCh38.p12.

### Differential expression analysis

Genes quantitation was calculated with the Cufflink software (version 1.3.0, 2010). The expression score of transcripts was calculated by the Cuffdiff package (version 2.2.1, 2013), as RPKM = total exon reads/mapped reads in millions × exon length in kb (RPKM = reads per kilobase of transcript per million mapped reads). The false discovery rate (FDR) was set as 5, and the q‐value (p‐adjusted) was given as <0.05.

### Clinical specimens

This investigation was performed with the approval of the National Institute of Genetic Engineering and Biotechnology (NIGEB) ethics committee (#971001-I-695). The study population consisted of 40 CRC and 40 lung cancer patients, who underwent cancer surgery at Shohadaye Tajrish Hospital, Tehran—Iran, during 2012–2018. Each participant received and filled an informed consent before sampling. Subjects median age was 65 years (range: 60–70 years). As for healthy control, a matched adjacent normal sample was also received from each patient. Samples were liquid nitrogen-frozen and kept at -80°C. Tumors' assessment was performed based on the American Joint Committee on Cancer (AJCC) guidelines [25].

### Western blot analysis

Tissue samples were lysed in a lysis buffer consisted of sodium dodecyl sulfate (SDS, 0.1%), Triton X‐100 (1%,), Tris‐HCl (100 mM, pH 7.4), sodium deoxycholate (0.5%), NaCl (150 mM), protease inhibitor phenylmethylsulfonyl fluoride (PMSF, 1mM), ethylenediaminetetraacetic acid (5mM), and glycerol (10%). After heating for 95°C for 5 min, the mixture was cooled on ice and centrifuged for 5 min at 14,000 rpm to collect the supernatant. Analysis of protein concentration was done by using Bradford's method [26]. Electrophoresis was carried on a 12% SDS‐polyacrylamide gel, and the separated proteins were then electrotransferred onto a nitrocellulose membrane by using at a constant voltage (20 V) for one h at 4°C. After transferring, the membranes were washed with the Tris Buffered Saline with Tween-20 (TBST-10X) at room temperature for 5 min, incubated with the blocking buffer consisted of PBS (1X), Tween‐20 (0.1%,), and skim milk (5%) for one h, and rewashed with the TBS buffer again. Primary antibody incubation was done for an overnight at 4°C with a 1:1000 dilution of the following antibodies: anti-CALU (4C6) Mouse mAb (#11991S), anti-Aurora A (D3E4Q) Rabbit mAb (#14475S), anti-Phospho-Aurora A (Thr288) (C39D8) Rabbit mAb (#3079S), anti-MCM2 Antibody (#4007S), anti-Phospho-MCM2 (Ser139) (D1Z8X) XP Rabbit mAb (#12958S), and anti-β-Actin Antibody (#4967S) as a loading control. The membranes were then washed with the TBST buffer for three times and incubated for one h at room temperature with a 1:2000 dilution of the Anti-Mouse IgG, HRP-linked Antibody (#7076S, for CALU), or 1:2000 dilution of the Anti-Rabbit IgG, HRP-linked Antibody (#7074S, for AURKA and MCM2). All antibodies were purchased from Cell Signaling Inc., USA. The blots were then rewashed with TBST buffer, and the immunoreactive proteins were monitored using the SignalFire ECL Reagent (#6883S, Cell Signaling Inc., USA). Proteins bands were analyzed using a Densitometry scanner and the ImageJ software (NIH, Bethesda, USA). The relative density of protein bands was determined by measuring the integrated density of each band after subtraction of X‐ray film background. Before the analysis of the ratio of phosphorylated/total for AURKA and MCM2 proteins, the density value of each band was normalized to β-Actin and then calculated.

### Statistical analysis

The Chi-square test and unpaired unequal variance t-test were performed to calculate the sex and age variables among the studied groups. The Mann-Whitney U test assessed the levels of the genes between groups. The receiver operating characteristic (ROC) curve analysis was performed to evaluate the diagnostic power of the genes panel. The area under the ROC curve (AUC) was constructed as an accuracy criterion for the examination of the candidate genes panel. Overall survival (OS) of patients was plotted with Kaplan–Meier survival analysis. All data are represented as the mean ± S.D. (Standard deviation). Statistical significance was achieved when *P* < 0.05 (*). All statistical analyses were performed with IBM SPSS Statistics software version 22 (IBM, USA).

## Results

### Identification of calumenin related diseases

To identify the calumenin related diseases, DisGeNET database was used with the keyword CALU. According to the results, the expression pattern of CALU isoform 1 containing the canonical sequence (UniPort identifier: O43852-1), was increased in 3 phenotypes (Mental process, neoplastic process, and pathologic function), and ten human diseases (Table 1). Among the identified diseases, seven diseases were categorized as Neoplastic Process, and two other diseases, including Arteriosclerosis and Cystic Fibrosis, were identified as Disease or Syndrome group. The neoplastic category diseases consisted of colon and lung cancers.

**Table 1:** DisGeNET analysis of calumenin correlated diseases.

Disease Name	Type	Semantic Type	Disease Class	Calumenin Status
**Arteriosclerosis**	disease	Disease or Syndrome	Cardiovascular Diseases	Upregulation
**Cystic Fibrosis**	disease	Disease or Syndrome	Congenital, Hereditary, and Neonatal Diseases and Abnormalities; Digestive System Diseases; Respiratory Tract Diseases	Upregulation
**Atherosclerosis**	disease	Disease or Syndrome	Cardiovascular Diseases	Upregulation
**Carcinoma of lung**	disease	Neoplastic Process	-	Upregulation
**Colon Carcinoma**	disease	Neoplastic Process	-	Upregulation
**Malignant neoplasm of lung**	disease	Neoplastic Process	Neoplasms; Respiratory Tract Diseases	Upregulation
**Adenocarcinoma of lung (disorder)**	disease	Neoplastic Process	Neoplasms; Respiratory Tract Diseases	Upregulation
**Non-Small Cell Lung Carcinoma**	disease	Neoplastic Process	Neoplasms; Respiratory Tract Diseases	Upregulation
**Malignant tumor of colon**	disease	Neoplastic Process	Digestive System Diseases; Neoplasms	Upregulation
**Primary malignant neoplasm of lung**	disease	Neoplastic Process	-	Upregulation
**Intelligence**	phenotype	Mental Process	Behavior and Behavior Mechanisms	Upregulation
**Neoplasm Metastasis**	phenotype	Neoplastic Process	Neoplasms; Pathological Conditions, Signs and Symptoms	Upregulation
**Neoplasm Invasiveness**	phenotype	Pathologic Function	Neoplasms; Pathological Conditions, Signs and Symptoms	Upregulation

### Visualization of the calumenin associated genes network

**Fig 1:**
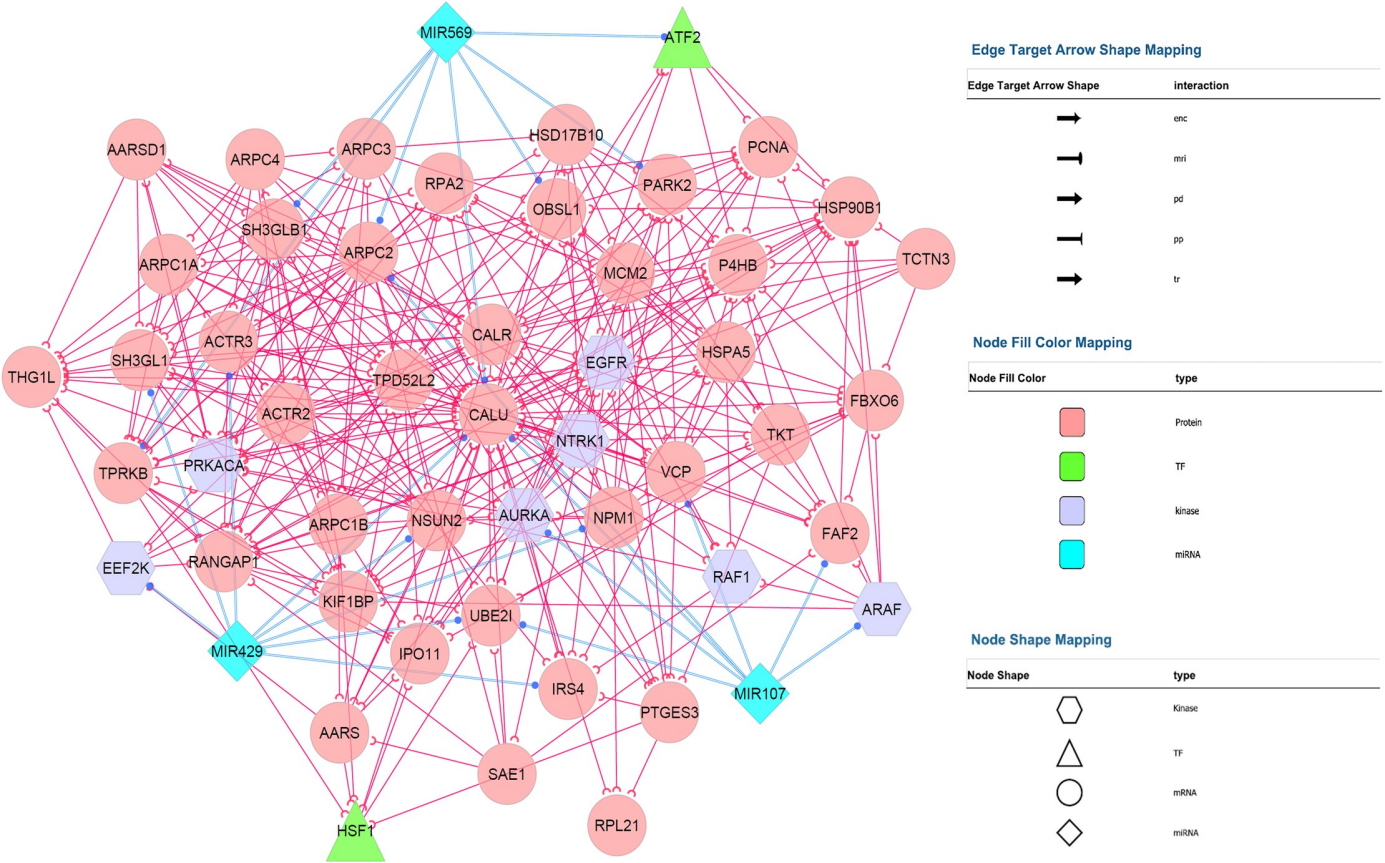
Construction of calumenin co-expressed genes network. The network was visualized using the Cytoscape plugins BisoGenet and CentiScaPe.

Calumenin associated gene network illustration was performed with the Cytoscape software. The molecular interaction between CALU and other genes was analyzed by the Cytoscape plugin BisoGenet, and the final network consisted of 127 nodes and 739 interactions. To create a subnetwork consisting of the key nodes, we used the CytoHubba algorithms and selected the hub objects (degree value >5) within the primary network. Fig 1 depicts the final system consisted of 52 high degreed nodes with 354 interactions.

In cellular biology, the term of the network represents a complex signaling mechanism consists of the essential information for a specific physiological action. Therefore, the analysis of a signaling system requires the use of graph-theoretic parameters, including centrality, motifs, etc., to depict the network organization. Like other kinds of directed networks, the nodes of the current system (Fig 1) were categorized based on their incoming and outgoing edges. Degree is the simplest type of the centrality measures and shows the importance of a node in the network based on its interaction (edge) with other nodes in directed networks. Using this classification, those nodes (top 1–5%) with the highest degree are termed as hubs and have an important role in the network. On the other hand, enrichment analysis of these nodes explains the physiological aspects of the target network. For example, the node representing the CALU protein in Fig 1, having an out-degree of 12 and an in-degree of 14, indicating the important role of this protein in the complex network existed between known cancer related signaling factors such as AURKA, EEF2K, EGFR, MCM2, RAF1, etc., which mediate the cancer cells proliferation and survival.

### Analysis of calumenin co-expressed genes perturbation in TCGA

The studied population consisted of 239 TCGA colon adenocarcinoma profiles (TCGA-COAD, 107 normal and 132 CRCs), and 245 lung adenocarcinoma profiles (TCGA-LUAD, 108 normal and 137 tumors). Each dataset was divided into a training set and a validation set. As for TCGA-COAD, the training set consisted of 47 healthy controls and 60 CRCs, and the validation set consisted of 60 normal datasets and 72 CRCs. On the other hand, the training set of TCGA-LUAD consisted of 37 healthy controls and 50 lung cancer datasets, and the validation set consisted of 71 normal and 87 lung cancer datasets. Descriptive details of colon and lung cancer patients are shown in Table 2 and Table 3, respectively.

**Table 2:** Clinical features of the colon cancer group.

Variable	Training set	Validation set	Clinical samples	*p*-value
**Healthy count (%)**				
** Sex**				0.137
** Male**	24 (51.06)	29 (48.33)	18 (45.00)	
** Female**	23 (48.93)	31 (51.67)	22 (55.00)	
** Age (year)**				0.27
** Mean + SD**	55 ± 4	55 ± 6	65 ± 3	
**Colorectal cancer count (%)**				
** Sex**				0.339
** Male**	30 (50.00)	40 (55.56)	18 (45.00)	
** Female**	30 (50.00)	32 (44.44)	22 (55.00)	
** Age (year)**				0.614
**Mean + SD**	63 ± 6	63 ± 9	65 ± 3	
**TNM stage**				<0.01
** I**	13 (21.67)	18 (25.00)	7 (17.50)	
** II**	15 (25.00)	13 (18.06)	13 (32.50)	
** III**	18 (30.00)	28 (38.88)	15 (37.50)	
** IV**	14 (23.33)	13 (18.06)	5 (12.50)	
**Healthy versus cancer (*p* value^2^)**				
** Sex**	0.841	0.728	-	
** Age**	<0.001	<0.001	-	

**Table 3:** Clinical features of the lung cancer group.

Variable	Training set	Validation set	Clinical samples	*p*-value
**Healthy count (%)**				
** Sex**				0.071
** Male**	18 (48.65)	38 (53.52)	23 (57.50)	
** Female**	19 (51.35)	33 (46.48)	17 (42.50)	
** Age (year)**				0.728
** Mean + SD**	55 ± 7	55 ± 7	65 ± 5	
**Lung cancer count (%)**				
** Sex**				0.331
** Male**	25 (50.00)	43 (49.42)	23 (57.50)	
** Female**	25 (50.00)	44 (50.58)	17 (42.50)	
** Age (year)**				0.725
**Mean + SD**	64 ± 5	64 ± 6	65 ± 5	
**TNM stage**				<0.01
** I**	10 (20.00)	17 (19.54)	5 (12.50)	
** II**	13 (26.00)	20 (22.99)	15 (37.50)	
** III**	21 (42.00)	29 (33.33)	12 (30.00)	
** IV**	6 (12.00)	21 (24.14)	8 (20.00)	
**Healthy versus cancer (*p* value^2^)**				
** Sex**	0.919	0.873	-	
** Age**	<0.001	<0.001	-	

**Fig 2:**
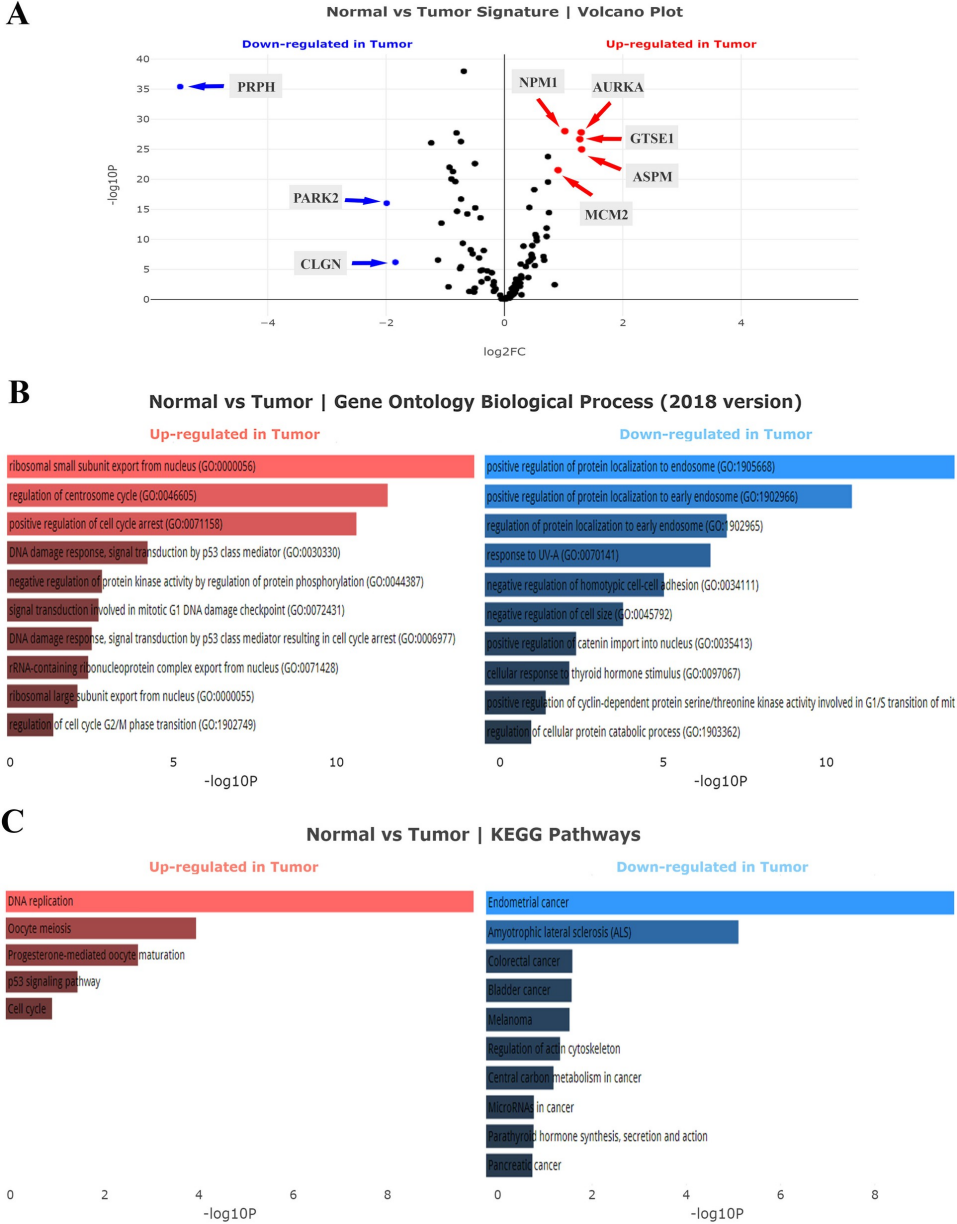
Analysis of calumenin correlated genes in TCGA-COAD. **(A)** Volcano plot analysis of the TCGA-COAD dataset. **(B)** Gene Ontology (GO) biological process analysis. **(C)** KEGG pathways analysis. The statistical significance of each gene was calculated using the Cuffdiff package and demonstrated as log2-fold changes in the volcano plot. Red points mean significantly upregulated genes, and blue points mean downregulated genes.

**Fig 3:**
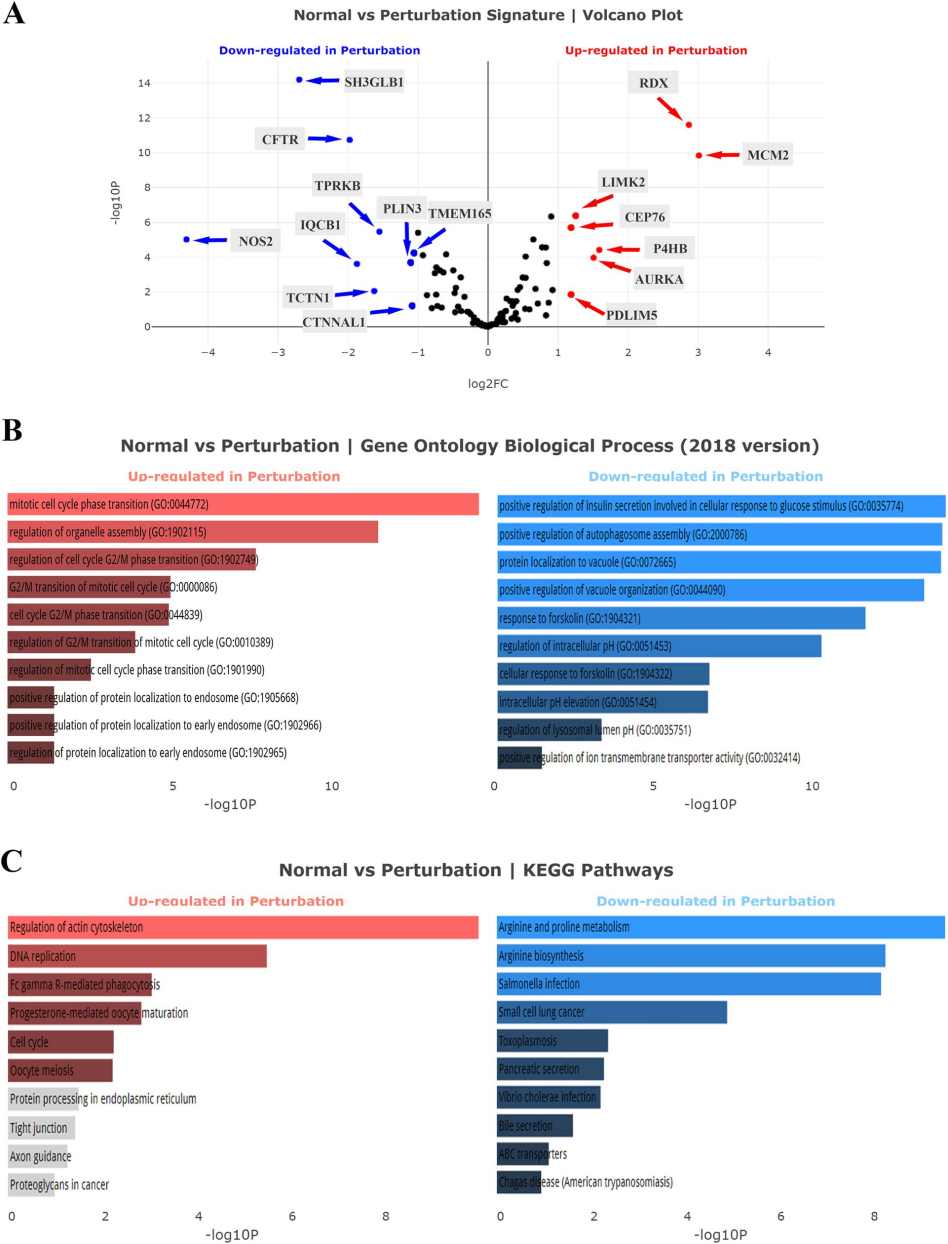
Analysis of calumenin correlated genes in TCGA-LUAD. **(A)** Volcano plot analysis of the TCGA-LUAD dataset. **(B)** Gene Ontology (GO) biological process analysis. **(C)** KEGG pathways analysis. The statistical significance of each gene was calculated using the Cuffdiff package and demonstrated as log2-fold changes in the volcano plot. Red points mean significantly upregulated genes, and blue points mean downregulated genes.

Following the data processing, DEGs of each cancer were analyzed using the cufflink and visualized with the Volcano plot. The complete list of upregulated and downregulated genes of TCGA-COAD and TCGA-LUAD datasets was presented in S1 Table, respectively. As already shown, the expression level of CALU was significantly upregulated in both colon adenocarcinoma and lung adenocarcinoma samples in comparison to the normal groups (*P* < 0.001). Volcano analysis of the TCGA-COAD dataset indicated PRPH, PARK2, and CLGN as significantly downregulated genes, and ASPM, AURKA, GTSE1, NPM1, and MCM2 as most significant upregulated genes (*P* < 0.001, Fig 2A). Meanwhile, analysis of TCGA-LUAD dataset introduced NOS2, SH3GLB1, CFTR, IQCB1, TCTN1, TPRKB, PLIN3, CTNNAL1, and TMEM165 as most downregulated genes along with MCM2, RDX, P4HB, AURKA, LIMK2, PDLIM5, and CEP76 as significantly upregulated genes (*P* < 0.001, Fig 3A).

### Gene set enrichment analysis

Gene set enrichment analysis (GSEA) is a statistical evaluation performed to identify the most significant biological terms in a given gene set, including signaling pathways, molecular functions, diseases, etc. Here, we used the Enrichr web-based application to display CALU co-expressed genes network enrichment results.

GO enrichment analysis of TCGA-COAD dataset showed that the upregulated genes were over-presented in GO biological process: ribosomal small subunit export from the nucleus (GO:0000056), and regulation of centrosome cycle (GO:0046605) (Fig 2B) while pathway enrichment analysis of these datasets indicated KEGG's DNA replication as significant term in TCGA-COAD (Fig 2C).

Enrichment analysis of TCGA-COAD downregulated genes showed that the genes were over-presented in GO biological process: positive regulation of protein localization to endosome (GO:1905668), and positive regulation of protein localization to early endosome (GO:1902966) (Fig 2B). While, pathway enrichment analysis of these datasets indicated KEGG’s Endometrial cancer, and Amyotrophic lateral sclerosis (ALS) as significant terms (Fig 2C).

GO enrichment analysis of TCGA-LUAD dataset showed that the LUAD upregulated genes were over-presented in GO biological process: mitotic cell cycle phase transition (GO:0044772), and regulation of organelle assembly (GO:1902115) (Fig 3B) while pathway enrichment analysis of this dataset indicated KEGG's Regulation of actin cytoskeleton as a significant term (Fig 3C).

Enrichment analysis of TCGA-LUAD downregulated genes showed that the genes were over-presented in GO biological process: positive regulation of insulin secretion involved in cellular response to glucose stimulus (GO:0035774), positive regulation of autophagosome assembly (GO:2000786), protein localization to vacuole (GO:0072665), and positive regulation of vacuole organization (GO:0044090) (Fig 3B) while pathway enrichment analysis of these datasets indicated KEGG's Arginine and proline metabolism, and Arginine biosynthesis as significant terms (Fig 3C).

### Estimation of DEGs diagnostic accuracy in cancer groups

Next, we performed a ROC curve analysis to estimate the diagnostic accuracy of DEGs in each cancer (Table 4). Analysis of COAD profiles showed that CALU along with ASPM, AURKA, CLGN, EEF2K, EGFR, GTSE1, HSPA4L, ILK, LIMK2, MAST3, MCM2, NPM1, PARK2, PLIN3, PRKACA, PRPH achieved AUC > 0.7 (*P* < 0.0001). On the other hand, analysis of LUADs indicated CALU along with AURKA, ASPM, GTSE1, NPM1, MCM2, HSPA4L, PRKACA, PLIN3, LIMK2, EEF2K, ILK, EGFR, MAST3, PARK2, and PRPH with AUC > 0.7 (*P* < 0.0001).

**Table 4:** Diagnostic performance of DEGs in TCGA-COAD and LUAD datasets.

**TCGA-COAD**						
**Gene**	**Sensitivity**	**Specificity**	**Youden’s index J**	**AUC**	***p* value**	**95% CI**
**CALU**	96.5	61.7	58.2	0.85	<0.0001	0.7863 to 0.9260
**ASPM**	94.63	65.96	60.59	0.89	<0.0001	0.8408 to 0.9570
**AURKA**	95.12	76.6	71.72	0.88	<0.0001	0.8203 to 0.9582
**GTSE1**	94.63	63.83	58.46	0.87	<0.0001	0.8130 to 0.9420
**NPM1**	92.68	85.11	77.79	0.93	<0.0001	0.8955 to 0.9767
**MCM2**	95.61	48.94	44.55	0.85	<0.0001	0.7901 to 0.9286
**HSPA4L**	75.12	48.94	24.06	0.71	<0.0001	0.6537 to 0.7800
**PRKACA**	93.17	74.47	67.64	0.87	<0.0001	0.8130 to 0.9312
**PLIN3**	80	55.32	35.32	0.77	<0.0001	0.7012 to 0.8393
**LIMK2**	90.73	68.09	58.82	0.83	<0.0001	0.7549 to 0.9076
**EEF2K**	82.44	74.47	56.91	0.83	<0.0001	0.7663 to 0.8995
**ILK**	72.2	65.96	38.16	0.77	<0.0001	0.7008 to 0.8407
**EGFR**	87.32	59.57	46.89	0.78	<0.0001	0.7179 to 0.8594
**MAST3**	92.2	70.21	62.41	0.86	<0.0001	0.7939 to 0.9274
**PARK2**	83.41	74.47	57.88	0.86	<0.0001	0.8120 to 0.9196
**PRPH**	93.17	85.11	78.28	0.89	<0.0001	0.8249 to 0.9602
**TCGA-LUAD**						
**Gene**	**Sensitivity**	**Specificity**	**Youden’s index J**	**AUC**	***p* value**	**95% CI**
**CALU**	86.46	64.86	51.32	0.76	<0.0001	0.6764 to 0.8585
**AURKA**	85.42	59.46	44.88	0.76	<0.0001	0.6664 to 0.8629
**MCM2**	84.38	64.86	49.24	0.79	<0.0001	0.7072 to 0.8812
**PCNA**	82.29	67.57	49.86	0.77	<0.0001	0.6793 to 0.8697
**IRAK1**	84.38	67.57	51.95	0.76	<0.0001	0.6584 to 0.8652
**P4HB**	82.29	72.97	55.26	0.76	<0.0001	0.6575 to 0.8639
**DBN1**	75	64.86	39.86	0.72	<0.0001	0.6315 to 0.8217
**PRKAR1B**	50	72.22	22.22	0.57	0.179	0.4739 to 0.6783
**PRKCDBP**	50	75	25	0.61	0.035	0.5210 to 0.7175
**LIMK2**	64.58	64.86	29.44	0.64	0.011	0.5382 to 0.7445
**WARS**	50	72.22	22.22	0.57	0.2	0.4721 to 0.6720
**CNTROB**	80.21	64.86	45.07	0.68	0.001	0.5722 to 0.7916
**MSN**	66.67	56.76	23.43	0.65	0.0065	0.5460 to 0.7591
**RDX**	90.63	51.35	41.98	0.74	<0.0001	0.6505 to 0.8417
**CTNNAL1**	78.13	62.16	40.29	0.68	0.0008	0.5794 to 0.7956

### Establishment of the predictive panel

To determine the risk of being diagnosed with cancer, we built a logistic model between normal controls and cancer datasets in the training set (n = 107 for COAD and = 87 for LUAD). The results indicated CALU, AURKA, MCM2 as significant predictors for both COAD and LAUD (Table 4). The ROC curve for COAD was constructed using the logit model of DEGs: Logit(p) = 19.3608–0.2534 X (CALU)– 0.1822 X (AURKA)– 0.2016 X (MCM2). For LUAD, ROC curve was constructed as follows: Logit(p) = 14.1828–0.1955 X (CALU)– 0.1811 X (AURKA)– 0.1857 X (MCM2).

**Fig 4:**
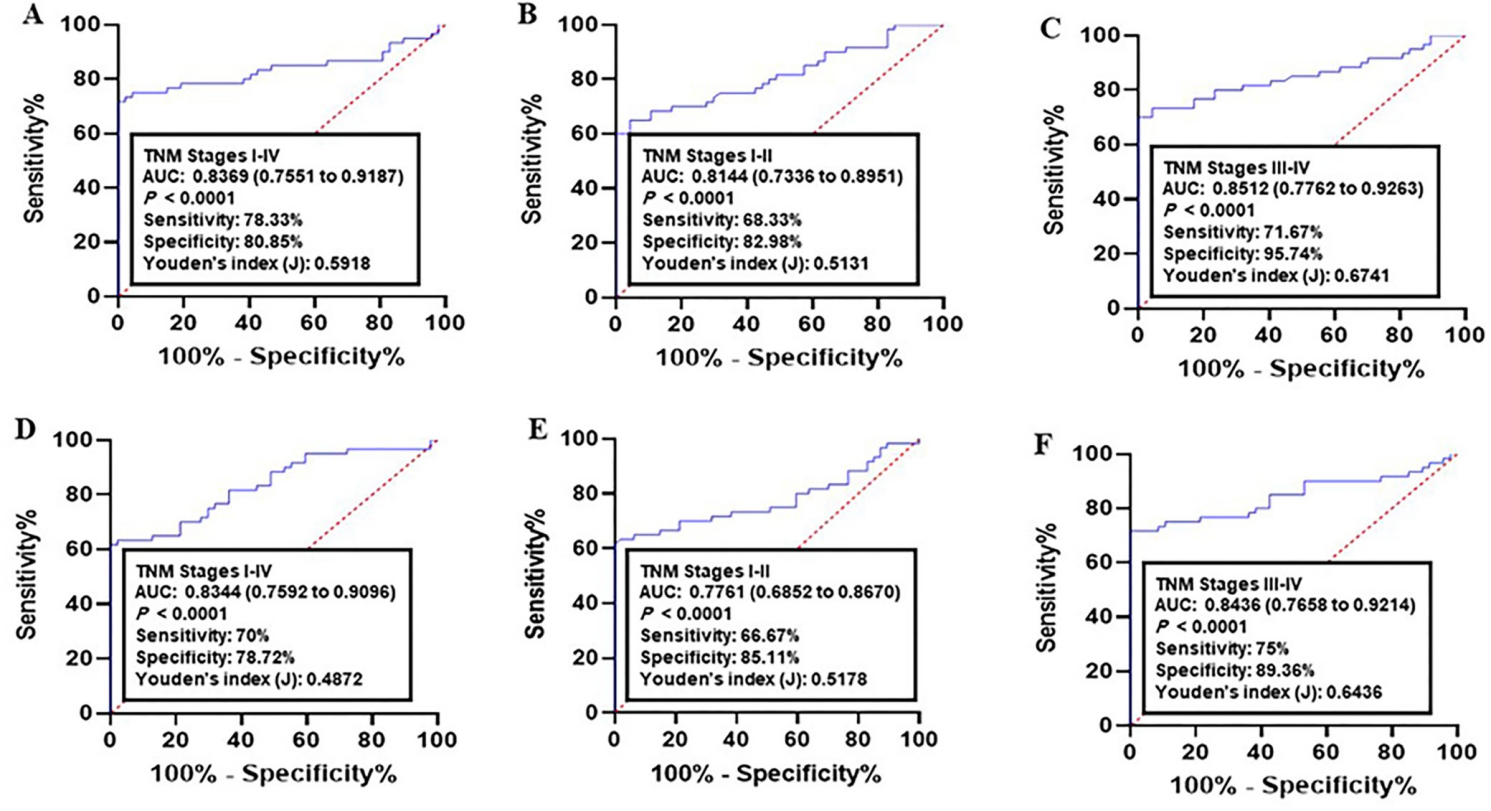
Receiver operating characteristics (ROC) curve analysis of the logit model with the CALU/AURKA/MCM2 panel. (A-C) The area under the ROC curve (AUC) assessment of the logit(p) value for the CALU/AURKA/MCM2 panel in distinguishing (A) colorectal cancer (CRC) patients (All TNM stages), (B) early CRC (I-II TNM stages), and (C) advanced CRC (III-IV TNM stages) from the healthy controls in training set. (D-F) The area under the ROC curve (AUC) assessment of the logit(p) value for the CALU/AURKA/MCM2 panel in distinguishing (D) CRC cases (All TNM stages), (E) early CRC (I-II TNM stages), and (F) advanced CRC (III-IV TNM stages) from the healthy controls in validation set. Logit(p) = 19.3608–0.2534 X (CALU)– 0.1822 X (AURKA)– 0.2016 X (MCM2).

Next, the diagnostic performance of established panel was estimated using the ROC data. Analysis of CRC samples versus normal group achieved an AUC of 0.8369 (95% CI = 0.7551–0.9187, 78.33% sensitivity and 80.85% specificity, Fig 4A). As for early CRC (I-II TNM stages), the AUC was 0.8144 (95% CI = 0.7336–0.8951; sensitivity = 68.33% and specificity = 82.98%, Fig 4B). Analysis of advanced CRC (III-IV TNM stages) resulted in an AUC = 0.8512 (95% CI = 0.7762–0.9263; sensitivity = 71.67% and specificity = 95.74%, Fig 4C).

Analysis of lung cancer datasets versus normal controls indicated an AUC of 0.8344 (95% CI = 0.7592–0.9096, 70% sensitivity and 78.72% specificity, Fig 4D). As for early cancer stages (I-II TNM stages), the AUC was 0.7761 (95% CI = 0.6852–0.8670; sensitivity = 66.67% and specificity = 85.11%, Fig 4E). Analysis of advanced cancer stages (III-IV TNM stages) resulted in an AUC = 0.8436 (95% CI = 0.7658–0.92.14; sensitivity = 0.75% and specificity = 89.36%, Fig 4F).

### Validation of the predictive panel

**Fig 5:**
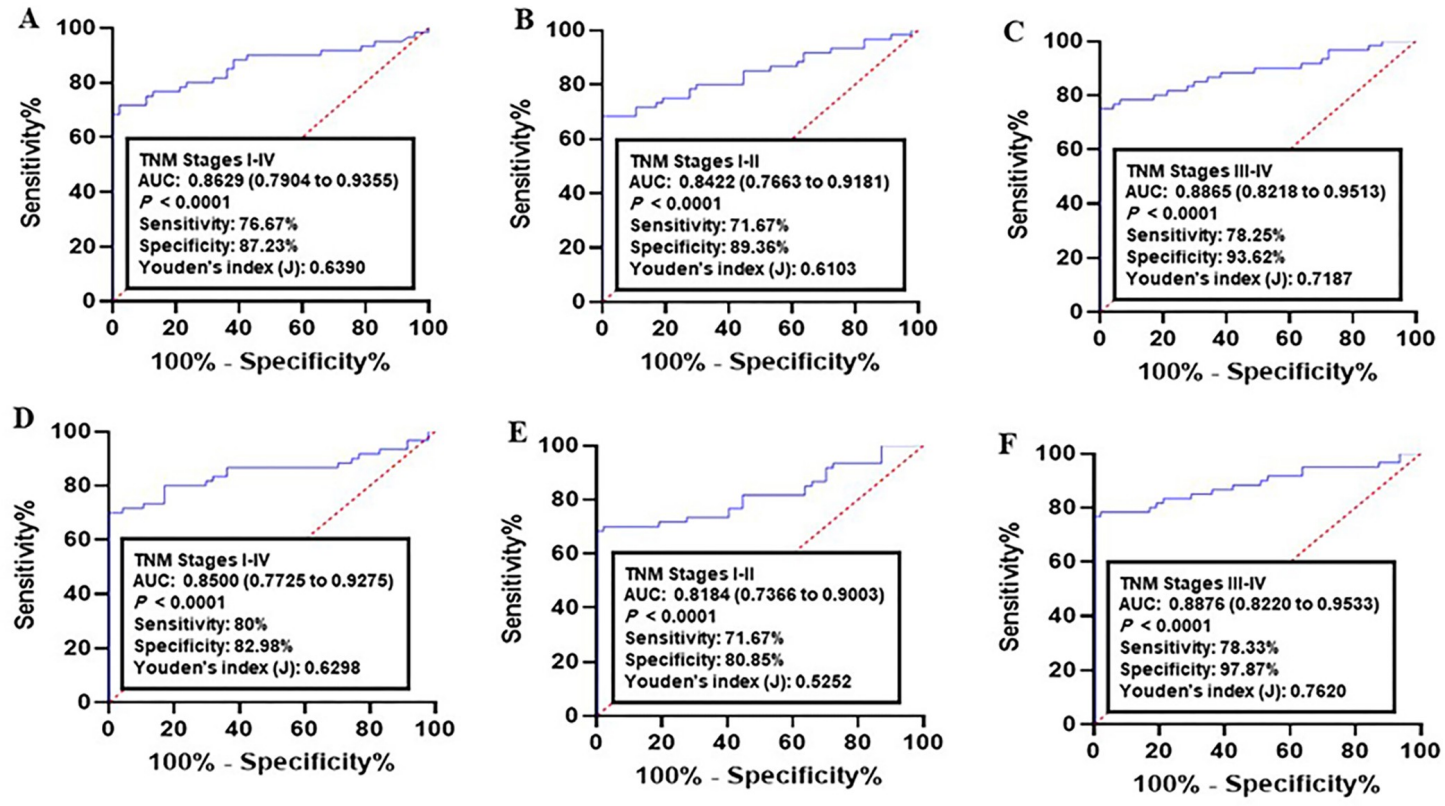
Receiver operating characteristics (ROC) curve analysis of the logit model with the CALU/AURKA/MCM2 panel. **(A-C)** The area under the ROC curve (AUC) assessment of the logit(p) value for the CALU/AURKA/MCM2 panel in distinguishing **(A)** lung cancer patients (All TNM stages), **(B)** early lung cancer (I-II TNM stages), and **(C)** advanced lung cancer (III-IV TNM stages) from the healthy controls in training set. **(D-F)** The area under the ROC curve (AUC) assessment of the logit(p) value for the CALU/AURKA/MCM2 panel in distinguishing **(D)** lung cancer cases (All TNM stages), **(E)** early lung cancer (I-II TNM stages), and **(F)** advanced lung cancer (III-IV TNM stages) from the healthy controls in validation set. Logit(p) = 14.1828–0.1955 X (CALU)– 0.1811 X (AURKA)– 0.1857 X (MCM2).

The diagnostic power of the panel was then validated in independent datasets (COAD: n = 132, and LUAD: n = 158). In COAD, the corresponding AUC for all stages (I-IV TNM stages) compared to normal group was 0.8629 (95% CI = 0.7904–0.9355; sensitivity = 76.67% and specificity = 87.23%, Fig 5A). Comparison of early CRC (I-II TNM stages) with normal achieved AUC = 0.8422 (95% CI = 0.7663–0.9181; sensitivity = 71.67% and specificity = 89.36%, Fig 5B). The AUC of advanced CRC (III-IV TNM stages) versus normal group was 0.8865 (95% CI = 0.8218–0.9513; sensitivity = 78.25% and specificity = 93.62%, Fig 5C).

As for LUAD, the corresponding AUC for all stages (I-IV TNM stages) compared to normal group was 0.8500 (95% CI = 0.7725–0.9275; sensitivity = 80% and specificity = 82.98%, Fig 5D). Comparison of early cancer (I-II TNM stages) with normal achieved AUC = 0.8184 (95% CI = 0.7366–0.9003; sensitivity = 71.67% and specificity = 80.85%, Fig 5E). The AUC of advanced lung cancer (III-IV TNM stages) versus normal group was 0.8876 (95% CI = 0.8220–0.9533; sensitivity = 78.33% and specificity = 97.87%, Fig 5F). These results indicated that the type of cancer or its status have no effect on diagnostic performance of the panel.

### Correlation between the abnormal levels of candidate genes and overall survival

**Fig 6:**
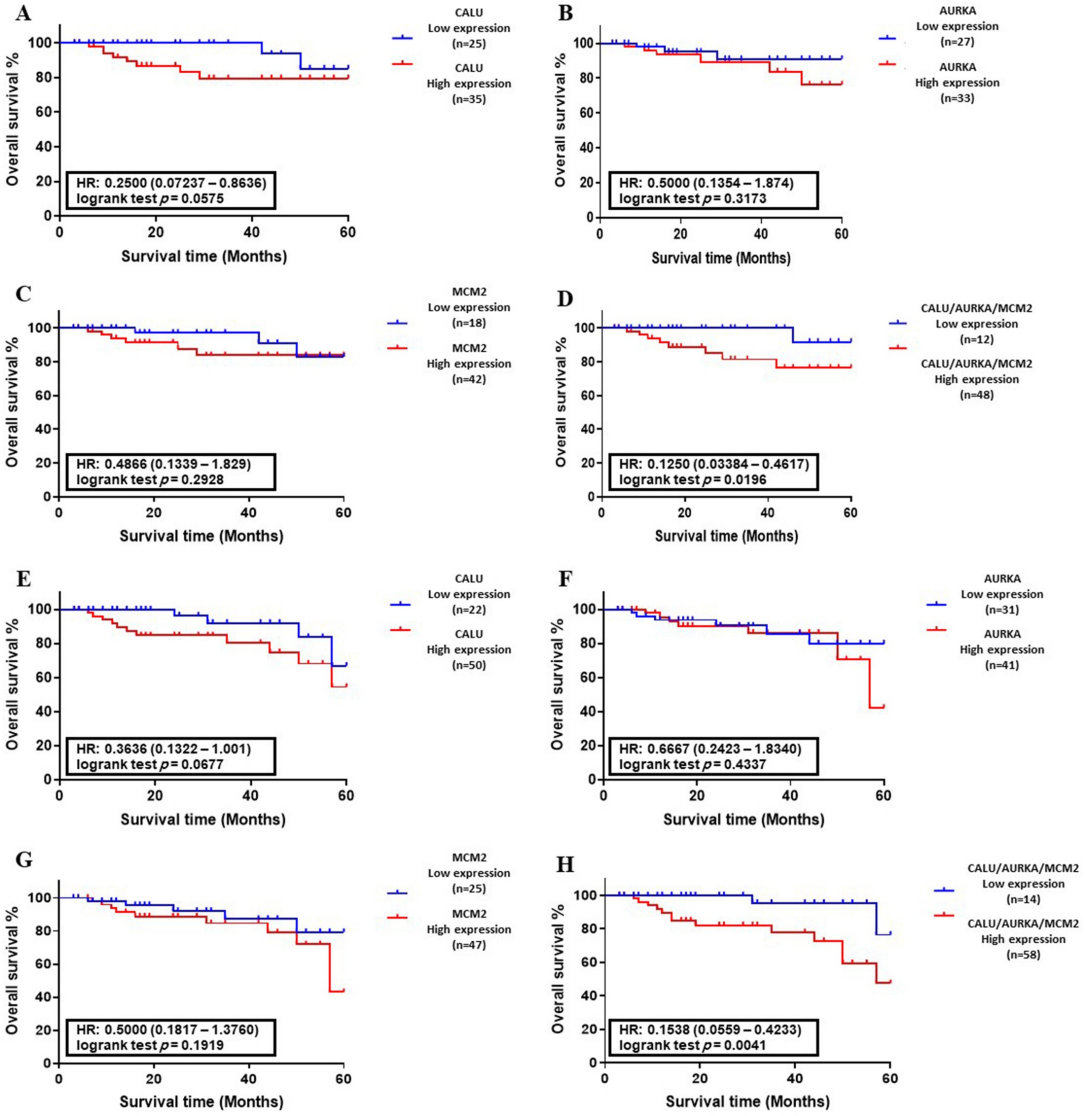
Association between DEGs expression and overall survival (OS) of colon cancer patients. **(A-D)** Kaplan–Meyer analysis of CALU, AURKA, MCM2, and CALU/AURKA/MCM2 panel in the training set (n = 107). **(E-H)** Kaplan–Meyer analysis of CALU, AURKA, MCM2, and CALU/AURKA/MCM2 panel in the validation set (n = 132). Data indicated that CALU, AURKA, and MCM2 in the form of a panel had a significant correlation with OS of colon cancer patients in training set (*P* < 0.05) and validation set (*P* < 0.01).

**Fig 7:**
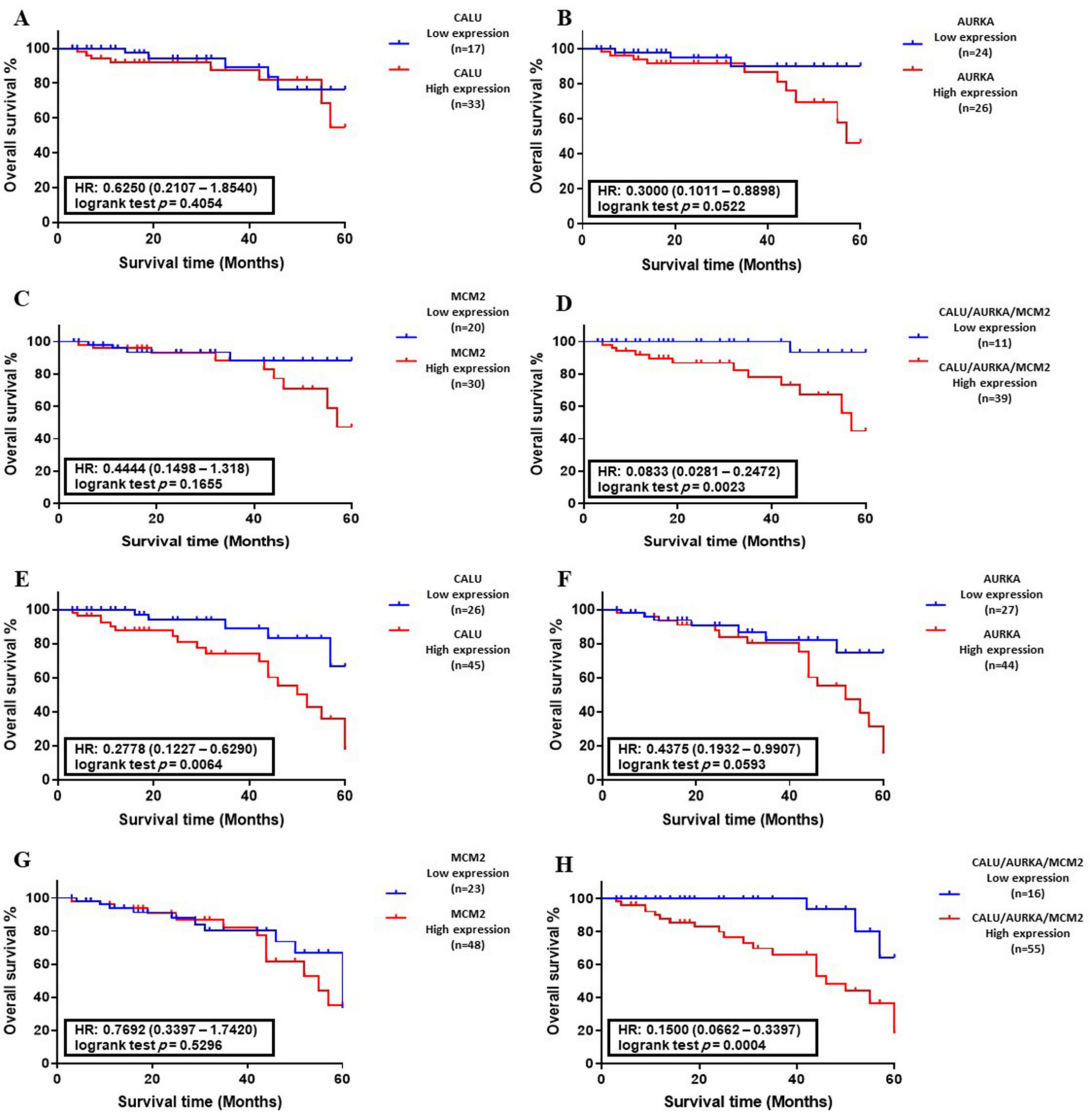
Association between DEGs expression and overall survival (OS) of lung cancer patients. **(A-D)** Kaplan–Meyer analysis of CALU, AURKA, MCM2, and CALU/AURKA/MCM2 panel in the training set (n = 108). **(E-H)** Kaplan–Meyer analysis of CALU, AURKA, MCM2, and CALU/AURKA/MCM2 panel in the validation set (n = 158). Data indicated that the abnormal level of CALU associated with the OS of patients with lung cancer (P < 0.01). Also, CALU, AURKA, and MCM2 in the form of a panel had a significant correlation with OS of lung cancer patients in training set (*P* < 0.01) and validation set (*P* < 0.001).

The Kaplan–Meier survival analysis was performed to plot the OS of colon and lung cancer patients with abnormal levels of CALU, AURKA, and MCM2, in separate form and as a panel. There was no significant correlation between DEGs level and OS of patients with colon and lung cancers in training sets (Fig 6A–6C and Fig 7A–7C). On the other hand, analysis of DEGs in validation sets indicated that the abnormal level of CALU associated with the OS of cancer patients with lung cancer (*P* < 0.01, Fig 7E).

OS analysis of CALU, AURKA, and MCM2 in form of a panel indicated a significant correlation between abnormal expression of DEGs and overall survival of CRC patients in both training set (*P* < 0.05, Fig 6D) and validation set (*P* < 0.01, Fig 6H), along with overall survival of lung cancer patients in training set (*P* < 0.01, Fig 7D) and validation set (*P* < 0.001, Fig 7H).

### Analysis of colon and lung cancer biopsy specimens indicated a gradual increment of CALU, AURKA and MCM2 proteins level through normal–carcinoma transition

To investigate the protein alteration of the candidate signature biomarkers through cancer progression, the protein level of CALU, AURKA, and MCM2 was analyzed between 40 biopsy specimens and matched adjacent healthy tissues collected from CRC and lung cancer patients. To eliminate the possible impact of the TNM stage parameter on the analysis results, each cohort was designed to have an equal number of early (I-II TNM stage, n = 20), and advanced (III-IV TNM stage, n = 20) cancer patients. Additional details are demonstrated in Table 2 and Table 3.

**Fig 8:**
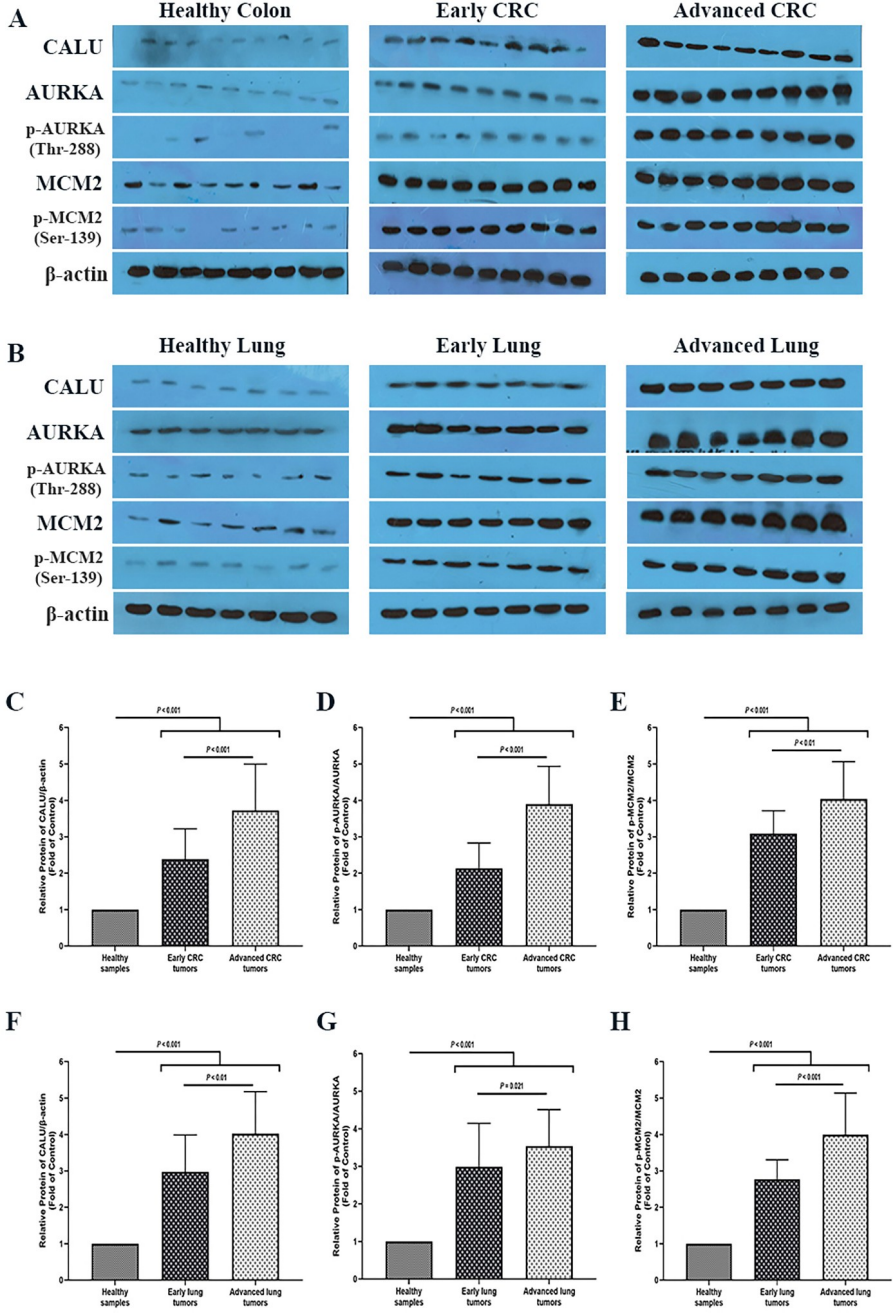
**A**nalysis of CALU, AURKA, and MCM2 proteins level in biopsy specimens and matched adjacent healthy tissues collected from **(A)** CRC and **(B)** lung cancer patients. **(C)** Comparative analysis of CALU level between healthy samples, early and advanced CRC tumors. **(D)** Comparative analysis of p-AURKA/AURKA ratio between healthy samples, early and advanced CRC tumors. **(E)** Comparative analysis of the p-MCM2/MCM2 ratio between healthy samples, early and advanced CRC tumors. **(F)** Comparative analysis of CALU level between healthy samples, early and advanced lung tumors. **(G)** Comparative analysis of p-AURKA/ AURKA ratio between healthy samples, early and advanced lung tumors. **(H)** Comparative analysis of the p-MCM2/ MCM2 ratio between healthy samples, early and advanced lung tumors. Western blot analysis was performed with a 1:1000 dilution of the anti-CALU antibody, anti-Aurora A antibody, anti-Phospho-Aurora A (Thr288) antibody, anti-MCM2 antibody, and anti-Phospho-MCM2 (Ser139) antibody. The quality of protein bands was measured with the NIH ImageJ software relative to the control band (B‐actin). Each value is the mean ± SD of six separate experiments. The asterisk indicates significantly different from the control group.

Western blot analysis of CALU, AURKA, and MCM2 proteins showed that the level of all of these candidate biomarkers was statistically increased through normal–carcinoma transition in both types of cancers (Fig 8). Comparative analysis of CALU between normal samples and all cancer tumors indicated a 3.2-fold and 3.63-fold enhancement of protein level in the CRC group and lung cancer group, respectively (*P* < 0.001). On the other hand, analysis of the CALU level between early and advanced tumor stages of CRC and lung cancer groups resulted in a 1.66-fold (*P* < 0.001), and 1.31-fold (*P* < 0.01) elevation of CALU level, respectively.

To investigate the alteration level of AURKA and MCM2 proteins, the ratio of their active form to their total level (phosphorylated AURKA at Thr-288 residue/total AURKA, and phosphorylated MCM2 at Ser-139/total MCM2) was analyzed between target groups. Comparative analysis of p-AURKA/AURKA between normal samples and all cancer tumors indicated a 3.43-fold and 3.12-fold enhancement of protein level in the CRC group and lung cancer group, respectively (*P* < 0.001). On the other hand, analysis of the p-AURKA/AURKA ratio between early and advanced tumor stages of CRC and lung cancer groups resulted in a 1.88-fold increment (*P* < 0.001), and 1.14-fold elevation (*P* = 0.021) of p-AURKA/AURKA ratio, respectively.

Similar results were obtained from the analysis of p-MCM2/MCM2 level between healthy samples and all tumor in CRC group (3.48-fold increment, *P* < 0.001), and lung group (3.56-fold increment, *P* < 0.00). Analysis of the p-MCM2/MCM2 ratio between early and advanced tumor stages of CRC and lung cancer groups resulted in a 1.27-fold increase (*P* < 0.01), and 1.44-fold elevation (*P* < 0.001) of p-MCM2/MCM2 ratio, respectively.

## Discussion

Tumor metastasis is defined as cancer cell migration from the primary site to distant locations and plays a critical role in cancer recurrence and mortality [27, 28]. As most of the current anticancer therapeutic efforts are focused on metastasis prevention, thus identification of metastasis signaling mediators is highly appreciated.

One of the main regulators of cancer metastasis is the extracellular matrix (ECM) proteins. ECM family consists of fiber molecules and proteins such as calumenin, which interact with extracellular focal adhesion complex (EFAC) and intracellular cytoskeleton, and through that improve the structural formation of cells [29]. Meanwhile, the positive impact of calumenin on cancer development was also reported [2, 8, 30–32]. According to the vital role of calumenin in tumor metastasis, it could be considered as a potential target for anticancer therapy. However, considering the lack of knowledge about the calumenin association with other signaling mediators, we tried to visualize and analyze calumenin co-expressed genes network in human cancers.

Analysis of CALU co-expressed genes network in TCGA datasets indicated that the network is markedly altered in human cancers. Diagnostic accuracy analysis of these DEGs in TCGA datasets COAD and LUAD showed that a gene panel that consists of CALU, AURKA, and MCM2 could successfully distinguish cancer tumors from healthy samples. Additionally, cancer cases with abnormal expression of these genes had a statistically meaningful lower survival rate than other patients.

AURKA is a kinase involves in destabilizing the microtubules of cancer cells by phosphorylation of the tumor suppressor RAS-association domain family 1, isoform A (RASSF1A), and subsequently increases abnormal cancer cells proliferation by disrupting the mitotic phase [33]. AURKA overexpression leads to consequent activation of the NF-κB signaling pathway via negative regulation of IκBα and induces tumor invasion [34]. One the other hand, AURKA plays a pivotal role in tumor survival by provoking Bcl-2 and MCL-1 [35, 36], and anti-apoptotic factors levels, blocking Bax, Bim, PUMA apoptotic mediators activity [35, 37, 38], along with disruption of the mammalian Target of Rapamycin (mTOR) autophagy pathway [38]. AURKA upregulation also suppresses cytochrome C releasing into the cytoplasm and prevents Apaf-1/cytochrome C apoptosome formation [39].

MCM2, the other member of our panel, belongs to the minichromosome maintenance protein complex family and has a critical impact on initiating DNA replication and proliferation of cells [40]. MCM2 is activated in response to growth signaling pathways and stimulates DNA replication, and therefore highly expressed in active proliferative cells, including cancer cells [41, 42]. Accordingly, an abnormal level of MCM2 has been reported widely to associate with human cancers, including breast [43], colon [44], gastric [45], rectal [46], and skin cancer development [47].

**Fig 9:**
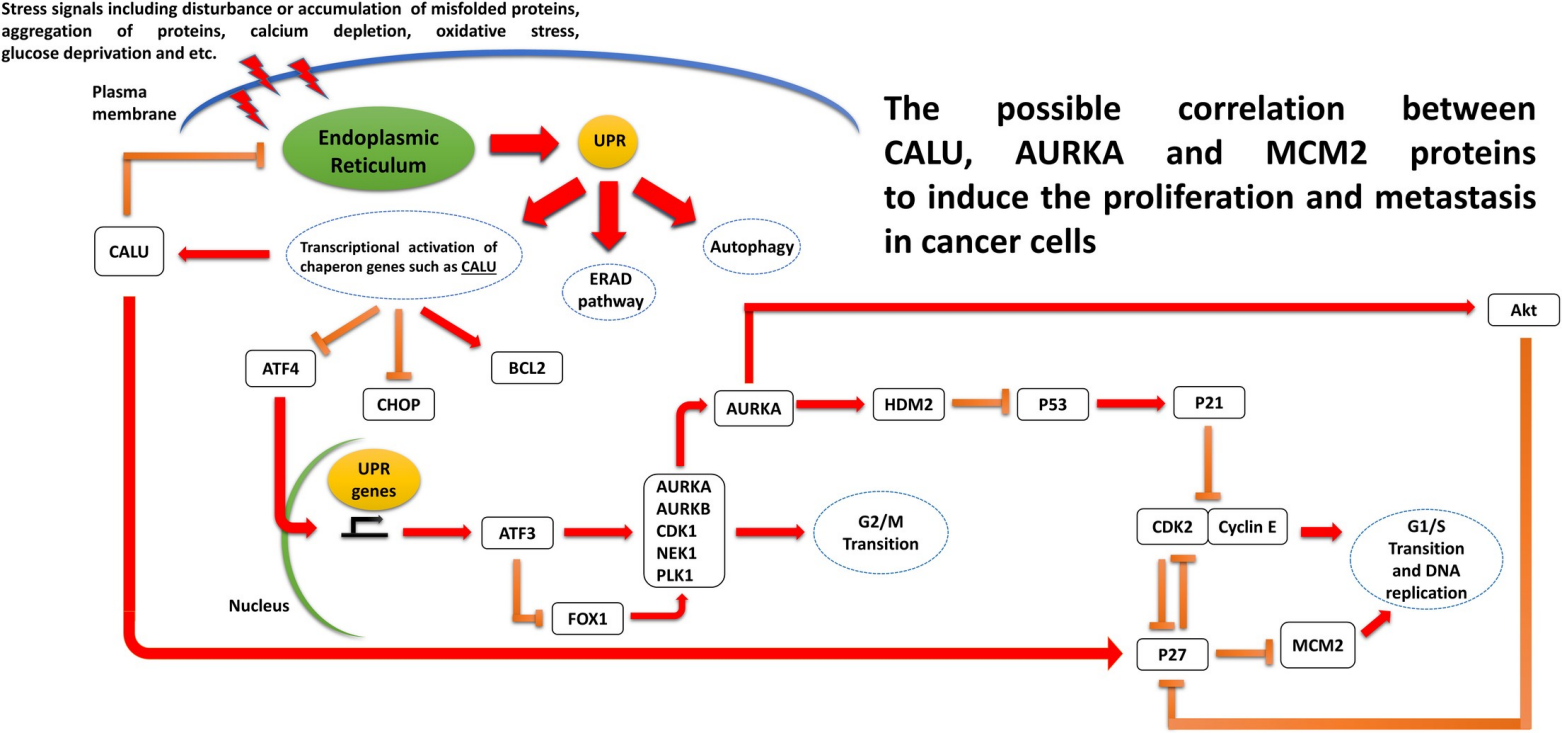
Schematic presentation of the correlation between CALU, AURKA, and MCM2.

To our knowledge, no investigation has been done to find the signaling interaction between CALU, AURKA, and MCM2 proteins. However, a survey in the published literature helped us to visualize the possible correlation between these candidate genes (Fig 9). As previously mentioned, the expression level of CALU is reported to be increased in advanced colon and lung cancer tumors [9, 11]. Likes other ER-resident chaperones, the expression of CALU can be triggered in response to ER stress and the unfolded protein response (UPR) caused by the physiological and pathologic stimuli such as metabolic disturbance, DNA damage, accumulation of misfolded proteins, calcium depletion, oxidative stress, and etc., which are directly associated with cancer initiation and development [48]. A recent study on neonatal rat cardiomyocytes showed that that the modulatory impact of CALU on ER stress is mediated thorugh the suppression of ER-initiated apoptosis markers CHOP, and p-JNK, alleviation of the UPR triggers pro-apoptotic PERK- ATF4 axis, and eventually increment of antiapoptotic protein Bcl-2 [49]. As a known UPR transcription factor gene, ATF4 is overexpressed under glucose deprivation and positively upregulates the ATF3 expression [50, 51]. It has been shown that ATF3 has a suppression impact on the mitotic kinase gene AURKA expression. The other negative effect of ER stress on AURKA expression can be mediated by AFT4, through the suppression of the FOXO1/AURKA expression axis [52]. AURKA is reported to remove the inhibitory impact of the p53/p21 axis on the activation of the Cyclin-dependent kinase 2 (CDK2), cell cycle transition, and tumorogenesis by promoting the HDM2-induced ubiquitination and inhibition of p53 [53]. This kinase also has a positive role in Akt activation to suppress the p27 protein, like the other negative regulator of CDK2 [53]. As a tumor suppressor, p27 is also reported to be able to block the G1/S transition by suppression of the MCM2 phosphorylation [54]. The suggested mechanism is illustrated in Fig 9. Although our analysis of the clinical samples of the CRC and lung cancer patients confirms the possible correlation, however, this theoretical mechanism needs to be investigated with the experimental validations to elucidate the exact underlying interactions.

Currently, there is a lack of a low-cost, noninvasive method with high sensitivity and specificity performances for cancer screening. For example, According to the American Cancer Society (ACS) screening guidelines for early detection of colon cancer [55], both men and women beginning at age 50 should follow one of the following testing schedules: (1) colonoscopy (every ten years); (2) flexible sigmoidoscopy (every five years); (3) double-contrast BE (every five years); or (4) CT colonography (every five years). Despite the high accuracy of these techniques, all the current screening methods have their own disadvantages. For example, using the colonoscopy as a golden standard for CRC screening requires unpleasant bowel preparation, and mostly is along with the pain experiment, discomfort, and embarrassment [56–58]. Therefore, a wide range of subjects delay their colonoscopy test or entirly avoiding it. Although other less-invasive screening methods such as fecal occult blood test (FOBT) and carcinoembryonic antigen (CEA) blood estimation are now using as alternatives; but, low sensitivity and specificity of these methods limit their use for cancer diagnosis [59–61]. Hence, detection of new biomarkers with more accurate diagnostic capability is needed for cancer screening in routine health checkups.

The current study suggests a multigene panel for cancer screening. Although a wide range of biomarker studies has been dedicated to a single gene exaination, this strategy is not always efficient. Some clinical features, such as the personal and familial history of patients, should be considered, especially in the case of hereditary diseases like cancer [62]. In addition, there is no guarantee that repeating a test for an individual gene will yield the initial results. Therefore, the application of a multigene panel for cancer diagnosis will save time and cost and can provide more accurate results. Besides that, it will elucidate the underlying interactions between candidate genes involved in malignancy.

In summary, analysis of CALU co-expressed genes network as one the key regulators in tumor metastasis introduced a gene panel that consists of CALU, AURKA, and MCM2 with high discriminative accuracy between healthy and cancer (colon and lung) populations. Also, abnormal levels of these genes had a reverse correlation with the patients' survival rate. This data indicates that the interactions between CALU, AURKA, and MCM2 has a pivotal role in cancer development, and thereby needs to be explored in the future.

## Supporting information

S1 Table The expression level analysis of upregulated and downregulated genes between tumors and healthy samples in TCGA-COAD (n = 107), and in TCGA-LUAD (n = 87) datasets in the training set. (XLS)
